# Supplementary material for: Association ankle function and balance in community-dwelling older adults
Source: PLoS One. 2021 Mar 4;16(3):e0247885. doi: 10.1371/journal.pone.0247885 (PMC7932177; doi:10.1371/journal.pone.0247885)
Supplement: Attachment — Submitted filename: Comments to authors_PONE-D-20-30676.docx [file pone.0247885.s003.docx]

**Comments to authors**

**Manuscript number**: PONE-D-20-30676

Thank you for sending me this manuscript. After thoroughly review it, some concerns would be clarified as follows:

Abstract

1. please add some magnitude of effects of the results the strongest predictor of dynamic balance.

Introduction

1. Please add other reasons for studying from a few of literatures evaluating relationship between kinematic and kinetic outcomes, and balance ability in the elderly. Does it have some conflicted evidence in this issue or what new information is added of this manuscript.

2. Change “goal” to “objective or aim” is more suitable.

Methods

1. Please provide information of these: study design, setting area of the study, recruitment method and period.

2. To include the eligible participants, do the participants should be active older adults?

3. Do other conditions such as neurological deficits DM with peripheral neuropathy, visual or auditory impairment or regular performing balance training strongly affect the outcomes of this study?

4. Please provide results of inter-rater reliability of two assessors.

5. Why do not the authors measure ankle inversion and eversion because such movements may influence balance ability in mediolateral direction of the elderly?

6. It seems that almost all parameters are lacking for details of testing session and representative values for statistical analysis, please add them.

7. Why do the authors only measure plantarflexors strength rather than measuring all ankle muscles?

8. It is required to add more information of balance testing that readers can do them accurately.

Discussion

1. Please give more reason why the results of the current study find that ankle mobility is a potential contributor to dynamic balance but not static balance.

2. I am not sure that sentences in Lines 225-227 are contrast together. Further, please provide more discussion of why plantarflexors strength show a high correlation with static balance.

3. According to lacking of other ankle movements such as inversion and eversion, the authors should note that the findings of this study would not cover the prediction of dynamic balance ability in mediolateral direction (may be a common direction of falling in the elderly).
