# Supplementary material for: Association ankle function and balance in community-dwelling older adults
Source: PLoS One. 2021 Mar 4;16(3):e0247885. doi: 10.1371/journal.pone.0247885 (PMC7932177; doi:10.1371/journal.pone.0247885)
Supplement: Attachment — Submitted filename: response to reviewers.docx [file pone.0247885.s005.docx]

**Manuscript number**: PONE-D-20-30676-R2

**Dear editor and reviewers**

First, we would like to thank the time spent in carrying out this second review of our manuscript. We have studied the comments and suggestions. They are reasonable, and we do believe that they will increase the quality of the manuscript if we address them correctly,

In the following lines we respond point by point the considerations and suggestions made by reviewers and the amendments made by the authors to address these in the manuscript.

The new submission includes a document with the manuscript in which all the changes introduced by the authors have been indicated with Word track changes, as well as another document with the final and clean version. This last version includes the corrections after English editing.

**Reviewer #1:** (No Response)

**Reviewer #2:**

The authors have revised the manuscript considerably, the resulting revised version has better clarity and is easier to read. The discussion section still has some language issues, and can be made more comprehensible with some more language editing.

- Page 10 line 221: “only predictive balance assessment according to resultant models” - Do the authors mean that this was the “only predictive factor of balance according to statistical models”? 🡺 That was the meaning indeed. The sentence has been amended.
- Page 10 line 222: “proposed ankle mobility measures only had a moderate association” - It is difficult to understand that the authors are talking about the low correlation between weight bearing and non weight bearing ROM because of the terms “proposed” and “association”. Remove “in fact”, and simply state that the “weight bearing and non-weight bearing ROM had only moderate correlation in our study, consistent with previous findings” 🡺 Ok, amended as suggested
- Page 10 line 223: “A possible explanation is that” - What does this explanation pertain to? The previous sentence talks about poor correlation between weight bearing and non weight bearing ROM, so the reader is expecting that the explanation is about that. As it is not, the authors have to state what the explanation is about at this point 🡺 The reviewer is right. We have changed the order of the sentences in this paragraph so that the ‘possible explanation’ is directly related to the previous sentence. The text is more clear now, thank you for noticing.
- Page 10 line 223-234: It is not easy to understand the “explanation”. Do the authors mean that weight bearing on the ankle is an important part of dynamic functional tasks, hence it is logical that weight bearing ROM becomes a determinant of dynamic balance? On the other hand, static balance depends on multiple other factors and not just ankle function, hence the role of ankle ROM in static balance is limited? Please re-write to allow the reader to follow the train of thought. 🡺 As all previous comments were related to the same paragraph, this has been rewritten for clarity. We hope it reads better now.
- Page 10 line 225: The authors mention static balance here along with dynamic, but in line 229, go on to say that ankle ROM is not important for static balance. This is contradictory and confusing 🡺 Indeed, this was a mistake that has been amended. As mentioned, the paragraph has been rewritten without losing the previous meaning.
- Page 10 line 231: “does not require a great ROM” - Do the authors mean “standing is not affected by minor ROM limitations at the ankle joint”? 🡺 Changed
- Page 11 line 247: “Increase in musculature” - Do the authors mean increase in muscle strength, power or muscle mass? 🡺 muscle strength. This has been corrected
- Page 11 line 248: “finding was only evident” – Do the authors mean that the relationship was present only when the eyes were open? 🡺 This was the case: changed
- Page 11 line 249: “implies greater confidence not only on balance” - Do the authors mean the elimination of vision results in greater reliance on proprioception for balance? 🡺 Corrected
- Please use simple and direct language for easy reading 🡺 The whole discussion section has been revised to address this comment, thank you for your review work.
